# First Isolation of *Yarrowia lipolytica* in a Granulomatous Pneumonia of a Spectacled Caiman, *Caiman crocodilus* Linnaeus, 1758

**DOI:** 10.3390/pathogens11111255

**Published:** 2022-10-28

**Authors:** Manuela Iurescia, Andrea Santini, Marco Montagnani, Elena Lavinia Diaconu, Fiorentino Stravino, Devid Agnelli, Emanuela Vergari, Gianluca Fichi, Claudia Eleni

**Affiliations:** 1 Istituto Zooprofilattico Sperimentale delle Regioni Lazio e Toscana, 00178 Roma, Italy; 2 Istituto Zooprofilattico Sperimentale delle Regioni Lazio e Toscana, 58100 Grosseto, Italy

**Keywords:** granulomatous pneumonia, *Candida pneumonia*, *Yarrowia lipolytica*, WGS, caiman

## Abstract

Contrary to humans, candidiasis is a rare infection in animals. However, in reptiles, candidiasis can cause gastrointestinal, cutaneous, or rarely systemic infections in stressed animals. The infections due to *Yarrowia lipolytica* have been increasingly described in human medicine, and hundreds of cases are reported, comprised of granulomatous lung lesions. Herein, granulomatous pneumonia of a spectacled caiman, *Caiman crocodilus*, was described, and the presence of *Y. lipolytica* in the lesion was confirmed through histopathology, microbiologic cultures, and molecular methods. The cause of death of the spectacled caiman was ascribed to bacterial shock septicemia consequentially to a traumatic lesion. However, in the right lung, several nodules containing white exudate were evidenced. At mycological and molecular analyses, *Y. lipolytica* was evidenced, and the histological finding confirmed the presence of a *Candida* infection in the lung granulomatous lesions. The comparison of ITS sequences with 11 *Yarrowia* spp. isolates, recently described in green sea turtles, and with a human strain was conducted, and the whole genome of a strain isolated in the spectacled caiman was sequenced. Even though *Y. lipolytica* is considered a non-pathogenic yeast and has been rarely described in animals, it seems to cause granulomatous lesions in reptiles as in humans.

## 1. Introduction

Despite disseminated candidiasis encompassing more than two-thirds of cases of fungal infections in human patients, *Candida* pneumonia is uncommon during invasive candidiasis, and the disease confined to the lungs in the absence of dissemination is rarer [1,2]. During *Candida* dissemination, the formation of granulomas is occasionally described in humans; nevertheless, patients can develop a pyogranulomatous inflammation in the liver, spleen, kidneys, and brain [3]. However, rare case reports of *Candida* granulomas in the lung have been described [4,5].

Contrary to humans, systemic *Candida* infection is usually rare in domestic animals, but it has been reported in several mammals (i.e., dogs, cats, sheep, calves, horses, ferrets, and alpacas) [6] and in birds [7,8]. In case of surgery or trauma, *Candida* can invade deeper tissue or the peritoneal cavity leading to peritonitis as reported in dogs [6], but a rare case of gastrointestinal candidiasis not associated with surgery or trauma has been reported in a cat [9]. To the best of our knowledge, *Candida* pneumonia in homoeothermic animals has never been reported, but lung infections have been evidenced during experimental infection in immunosuppressed mice [10]. In reptiles, candidiasis is not uncommon, and *Candida* can cause gastrointestinal, cutaneous, or rarely systemic infections in stressed animals [11,12]. Contrarily to other animals, *Candida* pneumonia has already been described in unique reports in several reptiles, such as crocodiles and caimans [12], and tortoises [13,14].

*Candida* species of major clinical importance in humans are *C. albicans* and some other non-albicans species, such as *C. glabrata*, *C. lusitaniae*, *C. krusei*, *C. kefyr*, *C. parapsilosis*, *C. tropicalis*, and less frequently *C. dubliniensis* [3]. Similarly, the most isolated species in mammal candidiasis are *C. albicans*, *C. glabrata*, *C. krusei*, *C. parapsilosis*, and *C. tropicalis*, while in bird crop candidiasis, *C. ravautii*, *C. salmonicola*, *C. guilliermondii*, *C. catenulata*, *C. brumptii, C. rugosa*, *C. famata*, *C. zeylanoides*, and *C. galli* have been also reported [15]. In reptiles, the most prominent cause of candidiasis is *C. albicans*; nevertheless, other non-albicans candidiases are rarely described due to *C. guilliermondii*, *C. palmioleophila,* and *C. tropicalis* [11].

*C. lipolytica* is the anamorph state of the newly named *Yarrowia lipolytica*, the widespread teleomorph state, largely studied as a model oleaginous yeast, and it is a frequent contaminant of dairy and meat products [16,17,18,19,20]. Its potential as a bio-control agent and eco-friendly bio-fertilizer in agriculture, as well as a promising platform for the eco-friendly production of chemicals and metabolites, has increased the research interest in the last years [21]. *Y. lipolytica* has also been largely studied as a source of animal food [16,19]. The American Food and Drug Administration (FDA) characterized *Y. lipolytica* as generally safe to use, and the European Food and Safety Authority (EFSA) declared it as a Novel Food safe for use in the general population over 3 years of age [16,19].

Despite this, from the first isolation in 1976 in ocular candidiasis, infection due to *Y. lipolytica* has been increasingly described in long-time catheterized persons, and hundreds of cases are reported in human medicine [18]. Among them, seventeen *Y. lipolytica* isolations were reported by Irby and colleagues (2014) from granulomatous lung lesions resected from patients suspected of malignant lung cancer. In animals, *Y. lipolytica* has been rarely reported, but recently it has been isolated in mild to moderate pneumonia and severe hepatitis in green sea turtles, *Chelonia mydas* [14].

Herein, we report for the first time granulomatous pneumonia due to *Y. lipolytica* in a spectacled caiman, *Caiman crocodilus* Linnaeus, 1758, confirmed through histopathology, microbiologic cultures, and molecular methods, and its whole genome sequence.

## 2. Materials and Methods

### 2.1. Case

A carcass of spectacled caiman, naturally deceased, was delivered to Istituto Zooprofilattico Sperimentale of Latium and Tuscany (IZSLT) for the investigation of the cause of death. The animal was died ten days before and conserved at −20 °C until it was thawed at 4 °C in the laboratory for the gross examination. The animal was an about 70-year-old male living in a recovery center in Grosseto province. The spectacled caiman had lived in the recovery center since 2015, in a terrarium (100 m^2^) with a 1 m deep pond supplied with natural spring water. The climate conditions were controlled through light and heating lamps, maintaining a temperature of 27 °C (minimum 24 and maximum 30 °C), but also openable windows were presented in the terrarium. The feeding was meat-based, and no signs of diseases or alterations were shown before its death. No other reptiles lived in the terrarium with the spectacled caiman or shared the same water.

### 2.2. Gross Examination

The animal was measured, following the method described by Britton and colleagues (2012), and examined for any external abnormality and post-mortem lesions. After dissection, internal organs were examined for any gross lesions, and immediately submitted for bacteriological and mycological examination. Feces were collected and conserved at 4 °C for parasitological examination. Samples of the lung, being suspected of pathological alteration, were collected and fixed in 10% neutral-buffered formalin for 48 h for histological examination.

### 2.3. Histological Examination

For histological examination, samples of the lung, previously fixed in formalin, were embedded in paraffin, and sections of 4 µm were routinely stained with Hematoxylin and Eosin (HE). Further sections were treated with Grocott-Gomori’s methenamine silver (GMS) or with periodic acid–Schiff (PAS) stains to enhance the detection of fungi within lesions.

### 2.4. Bacteriological Examination

Inoculums from the lungs, spleen, liver, kidney, and intracardiac clot were taken with a sterilized loop and plated onto Blood Agar and MacConkey Agar plates (IZSLT, Roma, Italy), and incubated at 25 °C for 36 h and at 37 °C for 24 h. Developed bacterial colonies were examined for taxonomical analyses according to Bergey’s Manual of Determinative Bacteriology [22]. Briefly, morphological characteristics of bacterial colonies, Gram staining, and oxidase and catalase results were considered, while biochemical reactions were performed on API test strips (Biomerieux ^TM^, Marcy-l’Etoile, France).

### 2.5. Parasitological Examination

During the post-mortem examination, parasitological external and internal investigations were conducted. Parasitological studies included a combination of gross examination, examination of organs using a dissecting microscope, screening of body fluids, and organ washes. Fecal samples were examined as briefly described. Two grams of samples were microscopically examined by flotation test with a low-density solution (saturated NaCl solution, specific gravity 1.200) and the other two grams with a high-density solution (ZnS solution, specific gravity 1.350).

### 2.6. Mycological Examination

Inoculums from the brain, lungs, spleen, liver, and kidney were taken with a sterilized loop and plated on Sabouraud Dextrose Agar and incubated at 25 °C for 7 days. The morphology of the colonies was examined for size, form, elevation, margin, texture, and color, while the microscopical examination was performed after Cotton Blue Lactophenol fresh staining through cell observation, including cell shape and budding. The biochemical identification was performed on ID 32C API test strips (Biomerieux ^TM^, Marcy-l’Etoile, France). In order to evaluate some virulence factors of the yeast strain, catalase and hemolytic activity was tested. Catalase activity was evaluated through the observation of releasing oxygen from hydrogen peroxide (H_2_O_2_) (Carlo Erba, Milano, Italy), while the hemolytic activity was measured following the method described by Abbes and colleagues (2017).

### 2.7. Molecular Analysis

For isolation of genomic DNA, yeast cell cultures were grown overnight at 30° in Yeast Extract–Peptone–Dextrose (YPD) broth to an early stationary phase before cells were harvested by centrifugation. Total genomic DNA was then extracted using E.Z.N.A.^®^ Yeast DNA Kit (Omega Bio-Tek Inc., Norcross, GA, USA) according to the manufacturer’s instructions. Extracted DNA was subjected to two PCR assays for amplification of the internal transcribed spacer (ITS) and D1/D2 ribosomal DNA regions [23,24]. The PCR protocols were performed using the primers ITS1-ITS4 and NL1-NL4, respectively (1.25 μL of each M13-tailed primer 10 pMol/μL) and the 1X KAPA HiFi Hot Start Ready Mix (Roche, Basel, Switzerland) kit. Then, 5 μL of DNA was added for the final volume of 25 μL. PCR started with an initial denaturation step of 3 min at 95 °C, followed by 35 cycles of 15 s at 95 °C, 15 s at 50 °C, 5 s at 72 °C and a final extension at 72 °C for 3 min. PCR products were purified using an enzymatic clean-up Exo-SAP-ITTM kit. Amplicons were Sanger sequenced on a 3500 Series Genetic Analyzer with BigDye Terminator chemistry (Applied Biosystems, Bedford, MA, USA) using M13-tailed primers. Sequence data analysis and trimming were performed using Geneious Prime software (version 2022 2.2). The resulting sequences were compared by BLAST analysis for isolate identification.

### 2.8. Library Preparation and Whole Genome Sequencing (WGS)

The isolate was analyzed in-depth by WGS. Library preparation was performed according to Alba and colleagues (2021) [25]. Briefly, libraries for short reads pair-end sequencing were prepared using the Illumina (Illumina, Inc., San Diego, CA, USA) NexteraXT R Guide 150319425031942 and sequenced on an Illumina platform (MiSeq sequencer, Illumina, Inc., San Diego, CA, USA).

### 2.9. Bioinformatics Analysis

Illumina raw reads were analyzed using an internal pipeline for assembly, which includes the following tools: FastQC (https://www.bioinformatics.babraham.ac.uk/projects/fastqc/, accessed on 25 September 2022), Trimmomatic [26], Spades [27] with the pipeline option: “—careful”) and Quast [28]. For FastQC, Trimmomatic and Quast were used as default parameters. Raw sequence data of our isolate (MOL9403) were submitted to the European Nucleotide Archive (http://www.ebi.ac.uk/ena, accessed on 25 September 2022) under study accession number ERR10295217. The whole genome of *Y. lipolytica,* herein isolated, was compared with publicly available *Y. lipolytica* whole genomes. In order to achieve a wider vision of overall genome relatedness, the average nucleotide identity (ANI) calculation was made by NCBI BLAST algorithm [29].

A phylogenetic analysis was performed comparing the ITS sequence obtained in this study with other ITS sequences of 11 *Yarrowia* spp. isolates recently described in green sea turtles [14] and one sequence of human origin (strain 1031_YLIP, GCA_001069125.1) [30]. Nucleotide (nt) sequence alignment was constructed using the ClustalW tool [31] with default parameters. The phylogenetic tree was built based on the Maximum Likelihood algorithm with the Tamura 3-parameter model +G [32] according to the best-fit substitution model, using the lowest Bayesian information criterion score. The bootstrapping probabilities were calculated using 1000 replicates. The tree was drawn to scale, with branch lengths measured in the number of substitutions per site. There was a total of 335 positions in the final dataset. Evolutionary analyses were conducted in MEGA X software [33]. 

## 3. Results

### 3.1. Gross Examination

**Figure 1:**
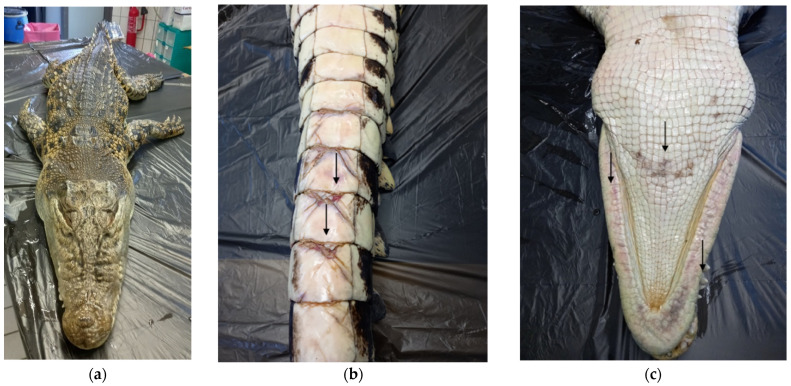
External examination of spectacled caiman (**a**) Dorsal picture of the carcass. (**b**) Ventral picture of the tail presenting mild rubbing injuries with mild hyperemia (arrow). (**c**) Ventral picture of the sub-mandibular area presenting mild rubbing injuries with mild hyperemia (arrow).

By the measurement of the animal, the total length (TL) was 2.51 m, and the dorsal cranial length (DCL) of 320 mm with a ratio of DCL to TL was 1:7.84. Snout–pelvis length (SPL) was 1.27 m, and tail length (TaL) was 1.24 m, with TaL to TL ratio of 1:1.02. The maximum head width (MHW) and the maximum cranial width (MCW) were respectively 200 mm and 100 mm, while snout–eye length (SEL) was 220 mm. At the external examination, the animal appeared in good nutritional body condition (Figure 1a). Only some mild rubbing injuries with mild hyperemia on the ventral face of the tail (Figure 1b), limbs, and sub-mandibular area were observed (Figure 1c). 

**Figure 2:**
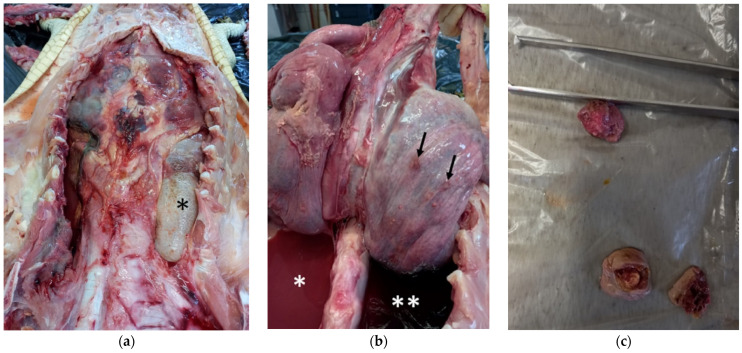
Internal examination of spectacled caiman (**a**) Right emphysematous lung (asterisk). (**b**) Left pleural cavity containing copious amount of pinkish-red purulent fluid (asterisk), right pleural cavity containing a lesser amount of dark red hemorrhagic fluid (double asterisks), and nodules visible on right lung surface (arrows). (**c**) Nodules from right lung after excision and cutting, showing a tick wall and containing white exudate.

At the internal examination, the left lung appeared collapsed, while the right lung was emphysematous (Figure 2a), and six macroscopically visible nodules around 1 cm of diameter were evidenced (Figure 2b) (description of the nodules in Supplementary Material). The left pleural cavity contained a copious amount of pinkish-red purulent fluid (Figure 2b), while the right pleural cavity contained a lesser amount of dark red hemorrhagic fluid (Figure 2b). After excision and cutting, the nodules showed a tick wall, sometime containing white exudate (Figure 2c).

The left lung presented a caudal laceration in continuity with the gastric wall. In the gastric cavity and in the pulmonary laceration, there was a pointed brown plastic foreign body of 34 cm in length and 10 cm of the base, which was positioned in the gastric cavity, while the tip was positioned in the pulmonary laceration. Inside the gastric cavity, a second foreign body of about 10 × 10 cm of the same material was found. Adhesions between the caudal wall of the left lung, the gastric wall, and surrounding organs that incorporate the spleen were observed. No other lesions were observed in internal organs and tissues.

### 3.2. Histological Examination

**Figure 3:**
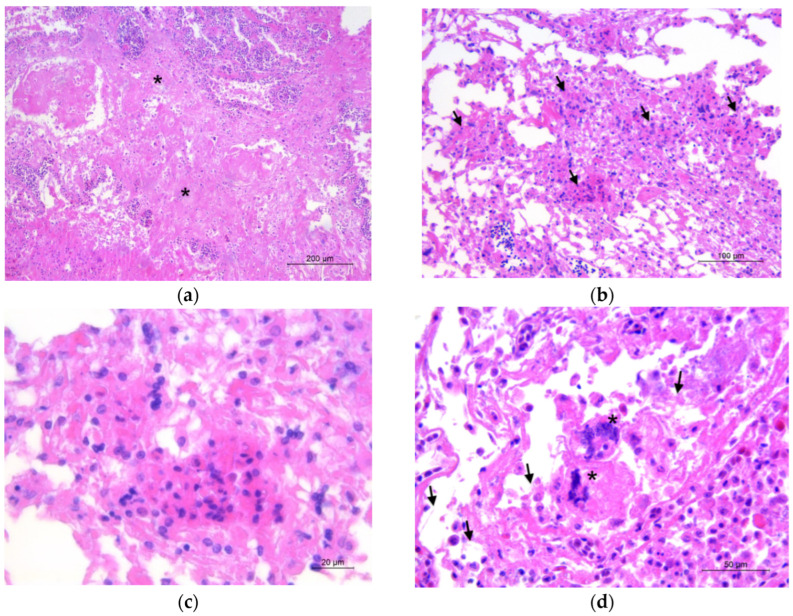

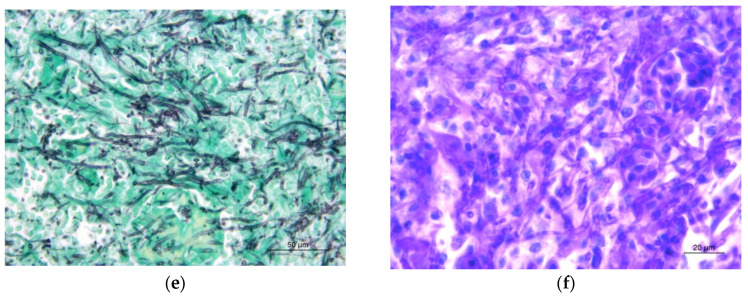
Lung. (**a**) Extensive coagulative necrosis (asterisks) in the parenchyma (HE; 10×). (**b**) Several scattered heterophilic granulomas (arrows) (HE, 20×). (**c**) High magnification of a heterophilic granuloma (HE, 63×). (**d**) Multinucleated cells (asterisks) and rare fungal hyphae (arrows) visible in the lung tissue (HE, 40×). (**e**,**f**). Numerous septate hyphae and spores were detected with GMS ((**e**); 40×) and PAS ((**f**); 63×) stains.

Microscopically, extensive areas of coagulative necrosis of the lung parenchyma and the pleural surface (Figure 3a) were observed, associated with the accumulation of fibrin and inflammatory infiltrate consisting predominantly of heterophils, lymphocytes, and macrophages. Scattered heterophilic granulomas (Figure 3b,c) with occasional multinucleated cells (Figure 3d) and rare fungal hyphae were seen. With GMS and PAS stains, numerous septate, occasionally branching hyphae and spores were diffusely detected in the necrotic tissue, within the granulomas, and in the bronchial spaces (Figure 3e,f). Vessels were frequently occluded by fibrin thrombi, and their walls showed vasculitis with accumulation of necrotic debris, fibrin, degenerate heterophils, and occasional fungal hyphae. Multiple small bacterial colonies were also observed in the lung parenchyma.

### 3.3. Bacteriological and Parasitological Examination

At the bacteriological examination, two bacterium species were isolated from the left lung, liver, spleen, and intracardiac clot. A Gram-negative rod shape bacterium produced nonpigmented smooth, round, grey, nonhemolytic colonies on Blood agar plates and colorless colonies, indicating non lactose fermenters (NLF) on MacConkey agar plates. It tested catalase positive and oxidase negative and was identified as *Serratia marcescens* through biochemical reactions on API test strips. The second Gram-negative rod shape bacterium tested catalase positive and oxidase negative and formed non-haemolytic, smarming colonies on Blood agar plates, while on MacConkey agar plates, the colonies were smooth, non-swarming and colorless (NLF). The API system identified it as *Proteus* spp. No parasites were evidenced at parasitological examination.

### 3.4. Mycological Examination

**Figure 4:**
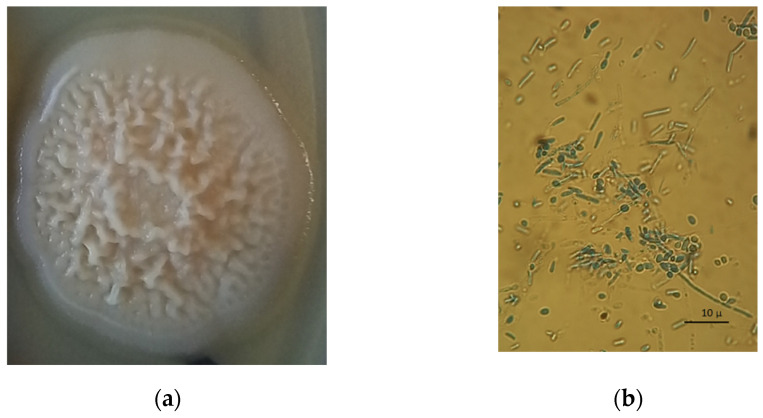
(**a**) White undulating colony presenting ridges, wrinkles, and uneven mounds. (**b**) Numerous septate hyphae and spores after Cotton Blue Lactophenol fresh staining 100×).

From mycological investigation, white undulating colonies presenting ridges, wrinkles, and uneven mounds were isolated from both lungs (Figure 4a). At the microscopic examination, a mix of hyphae and yeast cells was observed (Figure 4b). The API ID 32C gallery system identified the yeast as *Candida lypolitica*. The yeast strongly tested positive for catalase releasing oxygen from hydrogen peroxide (H_2_O_2_), and it showed weak hemolytic activity.

### 3.5. Molecular Analysis

Sequence data obtained by Sanger sequencing of the ITS region were then compared using BLAST (nucleotide collection nr/nt) and identified as *Yarrowia lypolitica*.

Overall, there are 27 publicly available *Y. lipolytica* whole genome assemblies [34]; however, eleven of them were discarded for the following reasons. The PO1f (two strains), YlCW001, FKP-355, and CLIB 122 strains (accession number respectively: GCA_009372015.1, GCA_000590845.2, GCA_009194645.1, GCA_003698155.1, and GCA_000002525.1) were discarded because they were engineered or mutants of wild type strains [35]. Three strains (GCA_001307795.1, GCA_001761485.1, and GCA_003054345.1) were complete genomes of the same strain (W29), while, for the DSM 3286 strain (GCA_014490615.1), was not possible to retrieve any related metadata. The strain NCIM 3590 (GCA_003571375.1) was discharged because previously identified as *Y. bubula* [36]. Finally, yet importantly, the unique sequence of human origin (strain 1031_YLIP, GCA_001069125.1) was not considered because it was not complete. Obtained ANI values for the remaining 16 sequences were reported in Table 1 as a percentage of alignment coverage and identity. Our sequence, MOL9403, shared with the other sequences a range of identity between 99.35 and 99.56% with a coverage range of 86.16–95.31%. The highest similarity was obtained with the XZ-2019 strain (Table 1) isolated from meat in China showing an identity of 99.49% with a coverage of 97.35% [37].

**Figure 5:**
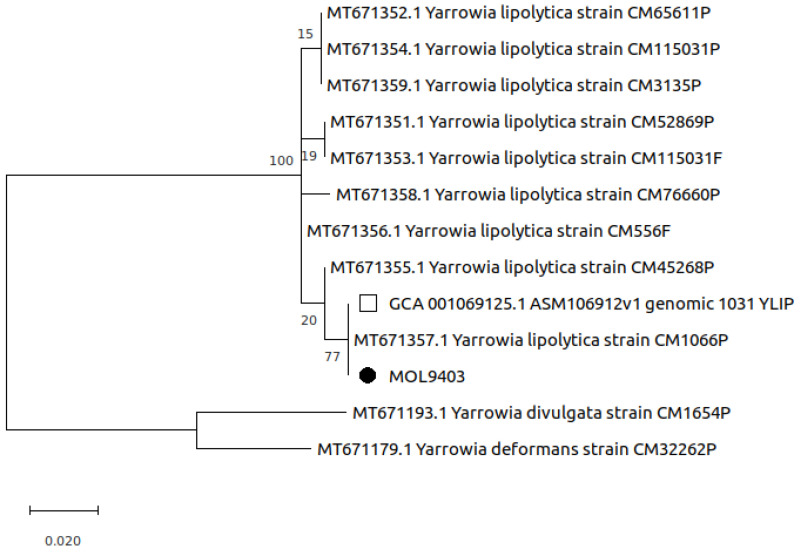
Phylogenetic analysis based on ITS sequence obtained in this study (MOL9403, black circle) with other ITS sequences of *Yarrowia* spp. isolates (green sea turtles) and one sequence of human origin (strain 1031_YLIP, empty square). The tree is drawn to scale, with branch lengths measured in the number of substitutions per site.

Phylogenetic analysis revealed the presence of two main groups of *Yarrowia* sequences. All the eleven *Y. lipolytica*, including the ITS sequence obtained in this study (MOL9403), were located in the largest group; these sequences differed only for one nucleotide to the sequence of the *Y. lipolytica* isolated from granulomatous lesions in sea turtles (strain CM1066P, MT671357.1) [14], and to ITS region extracted from the human origin sequence (strain 1031_YLIP, GCA_001069125.1) [30] The two remaining *Y. divulgata* and *Y. deformans* sequences clustered together in the smallest group (Figure 5).

## 4. Discussion

*Y. lipolytica* has been previously isolated in animals in epidemiological studies, such as on mycotic mastitis of cows [49], on fungal flora of the chicken combs [50], and on traumatogenic structures (i.e., stings, rays of fins, and sharp teeth) of several species of fish as human pathogenic fungus [51]. However, this yeast was also identified as the cause of oral lesions in cinereous vultures, *Gyps fulvus* [52], and in fungal dermatitis of two harbor seals, *Phoca vitulina* [53] (Table 2). In reptiles, this yeast has been reported in hepatic necrosis with infarctions of a Black-masked Racer, *Coluber constrictor latrun-culus*, where also *Edwardsiella tarda* was isolated from the liver (Table 2) [54]. In addition, recently, Domiciano and colleagues (2022), in a study to estimate the prevalence and types of granulomatous diseases in green sea turtles, *Chelonia mydas*, in Brazil, isolated *Y. lipolytica* in eight lungs with mild to moderate granulomatous pneumonia and in two livers with severe hepatitis of nine green sea turtles (Table 2) [14]. In two green sea turtles with *Y. lipolytica* infection, the cause of death was ascribed to septic shock [14]. In the present case, the cause of death of the spectacled caiman can be ascribed to *Serratia marcescens* and *Proteus* spp. shock septicemia consequentially to the traumatic lesion, while the granulomatous lesions in the lungs could be prior to the traumatic event. The emphysema observed in the right lung of the spectacled caiman could be vicariant emphysema due to the collapse of the left lung. However, mild multifocal emphysema was observed by Domiciano and colleagues (2022) in a green sea turtle with mild multifocal granulomatous pneumonia due to *Y. lipolytica* [14].

Even though many communications claim that *Y. lipolytica* is non-pathogenic, it would be more correct to consider it as a rare opportunistic fungal pathogen [21]. This yeast can cause candidemia in immunocompromised, antibiotic-treated, or critically ill patients through catheterization [21]. As a matter of fact, in human medicine, infections due to *Y. lipolytica* have been increasingly described in long-time catheterized persons, and 22 scientific reports describe *Y. lipolytica* clinical cases between 1976 and 2017 [18].

In human medicine, *Candida* pneumonia has been reported in severe clinical condition patients, such as immunocompromised individuals, infants with extremely low birth weight, and malignant tumor patients [2]. Particularly regarding *Y. lipolytica* pneumonia, in the study of Irby and colleagues (2014), this yeast was isolated from 24 patients suspected of malignant lung cancer, and among them, in 17 patients, it was isolated from resected lung granulomatous lesions [55]. None of the 24 patients had blood cultures positive for *Y. lipolytica*, and even if no patients were treated with antifungal therapy, no one developed fungemia during hospitalization [55]. With respect to other organs and tissues (skin, duodenum, mesentery, and breast tissue), the lung seemed to have the higher prevalence as site of *Y. lipolytica* isolation, and even if it was isolated from all five lobes, the upper lung lobe was observed as the preferred site of this yeast growth [55]. The isolation of *Y. lipolytica* from only one bronchoalveolar lavage (BAL) and benignity on granulomas pathological examination, as well as the absence of fungemia development, suggested to the authors that this microorganism could be considered as normal human flora capable of stimulating a granulomatous immune reaction [55]. 

However, the chronic granulomatous disease is characterized by infections with fungi producing catalase because they are not killed by neutrophils [56]. *Y. lipolytica* isolated in the present manuscript strongly tested positive for catalase. In addition, hemolysin was observed as an important virulence factor in *Y. lipolytica* strains [57]. In our isolate, low hemolytic activity was observed. However, in the study of Abbes and colleagues (2017), 22.40% and 12.00% of *Y. lipolytica* isolated from human patients with fungemia showed respectively weak and no hemolysis [57]. In the majority of research communications, *Y. lipolytica* is reported as non-pathogenic for humans; this is supported by the fact that most strains grow at temperatures lower than 32 °C [21]. In the case of reptilians, this evidence cannot exclude pathogenicity, as reptilians are not warm-blooded animals. However, in reptiles, captive environmental settings, such as humidity, temperature, excessive light or noise, and inadequate or unsuitable nutrition, generate acute or chronic stress that predisposes them to infections by opportunistic pathogens, such as fungi [58]. In crocodilians, fungal infections of the lungs are more common, such as in tortoises, than in other reptilians. In tortoises, opportunistic lung infections often occur secondary to deep trauma [59], while in captive crocodilians, mostly in crocodile hatchlings, *Paecilomyces lilacinus* and *Fusarium* pneumonia have been repeatedly described [58]. The stress of captivity, as well as the age, and not excluding the lung trauma and bacterial infection, could have created the condition for *Y. lipolytica* infection, as an opportunistic pathogen, in the spectacled caiman.

Due to the rarity of pneumonia not related to systemic Candidiasis in humans [2,4,60], the recent medical literature agrees that the diagnosis of *Candida* pneumonia must be confirmed by histopathology [1,61]. However, very few reports of *Candida* pneumonia in human patients have been confirmed through histopathology, microbiologic cultures, and molecular methods [2,60]. In our report, as well as in the study conducted by Domiciano and colleagues (2022) on green turtles, granulomatous pneumonia was confirmed by all three methods [14]. In addition, according to Meena and Kumar (2021), the only method to confirm the diagnosis of *Candida* pneumonia should be the demonstration of yeast or hyphae along with inflammatory cells in lung tissue. At the histological examination of the lung, we observed heterophilic granulomas with macrophages and multinucleated cells and numerous hyphae and spores within the granulomas. In reptiles, the hallmark of the inflammatory response is the granuloma, and the ‘heterophilic’ one is associated with extracellular pathogens (i.e., bacteria and fungi) [14].

To the best of our knowledge, the genome of our strain represents the first complete genome of a strain isolated from a clinical case in an animal. The complete sequences of *Y. lipolytica* isolates of animal origin are usually not available for comparison, as only the sequences of specific target genes (e.g., ITS, 244–453 bp) are publicly available so far. In this regard, our ITS sequence differed only for one nucleotide from the *Y. lipolytica* sequences obtained from granulomatous lesions previously described in green sea turtles (strain CM1066P, MT671357.1). The comparison of the whole genome of the two strains could be very interesting.

As a consequence of the great industrial interest in *Y. lipolytica*, as one of the most attractive non-conventional yeast, for a large range of white biotechnology applications, many molecular and genomic engineering studies have been conducted on this organism [35]. Among them, several authors sequenced the genome of wild-type, mutants, or engineered strains of *Y. lipolytica* [35], and a study on the pan-genome of this yeast was also conducted [62]. Despite these facts, similarly to what has been stated above on publicly available genomes of animal origin, there is a lack of complete genome sequencing of *Y. lipolytica* human isolates. Although the unique publicly available sequence obtained from a human clinical isolate [30] differed only for one nucleotide to our ITS sequence, we were not able to compare their whole genomes. 

## 5. Conclusions

Even though *Y. lipolytica* is considered the normal human mycobiota [53,55], this yeast is also seen as an opportunistic emerging pathogen, being responsible for cases of catheter-related candidemia [21]. In addition, as reported by several authors [14,47,50,52], *Y. lipolytica* could be responsible, alone or in combination with other pathogens, for animal infections. In the present manuscript, granulomatous pneumonia of a spectacled caiman was described, and the presence of *Y. lipolytica* in the lesion was confirmed through histopathology, microbiologic cultures, and molecular methods. Even though following Koch’s postulates, the pathogenicity of *Y. lipolytica* in reptilians cannot be confirmed without an experimental infection, all findings seem to confirm *Y. lipolytica* as the etiological agent of granulomatous pneumonia described. In addition, we reported the first genome sequence of a clinical isolate of this yeast in an animal. Not excluding the risk of animal–human transmission, the analyses and comparison of the complete genome of human and animal clinical isolates of *Y. lipolytica* could be notably interesting in identifying common specific genetic markers (e.g., virulence genes).

## Figures and Tables

**Table 1:** ANI values as percentage of alignment coverage and identity of 16 strains with MOL9403 sequence of *Yarrowia lipolytica* isolated in the spectacled caiman.

Assembly	Alignment Coverage	Percentage Identity	BioSample	Origin	State	Reference
	%	%				
GCA_023374015.1	97.359	99.492	XZ-2019	Meat	China	[37]
GCA_003571385.1	97.286	99.508	NCIM 3589	Marine strain	India	[38]
GCA_003367945.1	97.209	99.495	YB-566	N.R.	USA	[39]
GCA_900537225.1	97.165	99.489	H222	Soil	Germany	[40]
GCA_003367845.1	97.149	99.493	YB-567	Gluten settling water	USA	[39]
GCA_900087985.1	97.113	99.497	A101	Carwash effluent	Poland	[41]
GCA_000613145.2	96.974	99.445	WSH-Z06	Oil-polluted soil	China	[42]
GCA_020826875.1	96.876	99.511	CGMCC7326	Honeycomb	China	[43]
GCA_003367925.1	96.561	99.445	YB-419	Maize fiber tailings	USA	[39]
GCA_003054365.1	96.121	99.414	IBT 446	Feta cheese	Denmark	[44]
GCA_003243785.1	95.874	99.493	CBA6003	Kimchi	South Korea	[45]
GCA_023272835.1	95.401	99.475	22301-5	Waste waters	France	[46]
GCA_003367965.1	95.315	99.350	YB-420	Milled corn	USA	[39]
GCA_003054305.1	95.174	99.439	H222 (CLIB 80)	Soil	Germany	[47]
GCA_003367865.1	86.806	99.557	YB-392	Gluten settler	USA	[39]
GCA_012654145.1	86.162	99.568	W29	Waste waters	France	[48]

N.R.: Not reported.

**Table 2:** Isolation of *Yarrowia lipolytica* in animals.

Animal	Number of *Y. lipolytica* Positive/Number of Animals Tested	Origin of Isolation	Country	Lesions	Reference
Harbor seal, *Phoca vitulina*	2/16	1 swab and 1 skin scraping of skin	Tennessee, USA	Fungal dermatitis in facial-periorbital in one animal, not reported in the other	[53]
Fish (corvina or freshwater silver croaker, *Plagioscion squamosissimus*, piranha, *Serrasalmus maculatus*, dogfish, *Acestrothychus lacustris*, mandijuba or mandiamarelo catfish, *Pimelodus maculatus*, tilapia, *Tilapia* spp., and wolf fish, *Hoplias malabaricus*)	N.R.	Traumatogenic structures (stings, rays of fins, and sharp teeth)	Brazil	N.A.	[51]
Cinereous vultures, *Gyps fulvus*	3/6 nestlings	Scraped oral mucosa	Spain	Oral lesions	[52]
Cow	5 /484 cows with mastitis	Milk	China	Mastitis	[49]
Black-masked Racer, *Coluber constrictor latrunculus*	1/3	Liver	Louisiana, USA	Hepatic necrosis	[54]
Green sea turtle, *Chelonia mydas*	9/270	Granulomas from lung and liver	Brazil	Mild to moderate granulomatous pneumonia and severe hepatitis	[14]

N.A.: Not applicable.

## Data Availability

Not applicable.

## Supplementary Materials

The following supporting information can be downloaded at: https://www.mdpi.com/article/10.3390/pathogens11111255/s1, Table S1: Description of the six macroscopically visible nodules evidenced in the right lung of the spectacled caiman.
