# Supplementary material for: First Isolation of Yarrowia lipolytica in a Granulomatous Pneumonia of a Spectacled Caiman, Caiman crocodilus Linnaeus, 1758
Source: Pathogens. 2022 Oct 28;11(11):1255. doi: 10.3390/pathogens11111255 (PMC9698808; doi:10.3390/pathogens11111255)
Supplement: Supplementary file 1 [file pathogens-11-01255-s001.zip › pathogens-1979097-supplementary.pdf]

Table S1: Description of the six macroscopically visible nodules evidenced in the right lung of the spectacled caiman

Number	Diameter in cm	Description
1	1,5	The nodule presented a thick white wall and an increased consistency respect the lung tissue. After cutting, internally a white material surrounding by reddish tissue and a clear fluid were evidenced.
2	1,3	The nodule presented a thick white wall and an increased consistency respect the lung tissue. After cutting, internally a white material surrounding by reddish tissue and a clear fluid were evidenced.
3	1,2	The nodule presented a thick white wall and an increased consistency respect the lung tissue. After cutting, internally a reddish tissue was evidenced.
4	1,2	The nodule presented a thick white wall and an increased consistency respect the lung tissue. After cutting, internally a reddish tissue was evidenced.
5	0,9	The nodule presented a reddish wall and an increased consistency respect the lung tissue. After cutting, internally a reddish tissue was evidenced. The tissue of the lung surrounding the nodule was reddish.
6	0,8	The nodule presented a reddish wall and an increased consistency respect the lung tissue. After cutting, internally a reddish tissue was evidenced. The tissue of the lung surrounding the nodule was reddish.
